# Tungsten filled 3D printed field shaping devices for electron beam radiation therapy

**DOI:** 10.1371/journal.pone.0217757

**Published:** 2019-06-19

**Authors:** Lawrie Skinner, Benjamin P. Fahimian, Amy S. Yu

**Affiliations:** Department of Radiation Oncology, Stanford University, Palo Alto, CA, United States of America; Northwestern University Feinberg School of Medicine, UNITED STATES

## Abstract

**Purpose:**

Electron radiotherapy is a labor-intensive treatment option that is complicated by the need for field shaping blocks. These blocks are typically made from casting Cerrobend alloys containing lead and cadmium. This is a highly toxic process with limited precision. This work aims to provide streamlined and more precise electron radiotherapy by 3D using printing techniques.

**Methods:**

The 3D printed electron cutout consists of plastic shells filled with 2 mm diameter tungsten ball bearings. Five clinical Cerrobend defined field were compared to the planned fields by measuring the light field edge when mounted in the electron applicator on a linear accelerator. The dose transmitted through the 3D printed and Cerrobend cutouts was measured using an IC profiler ion chamber array with 6 MeV and 16 MeV beams. Dose profiles from the treatment planning system were also compared to the measured dose profiles. Centering and full width half maximum (FWHM) metrics were taken directly from the profiler software.

**Results:**

The transmission of a 16MeV beam through a 12 mm thick layer of tungsten ball bearings agreed within 1% of a 15 mm thick Cerrobend block (measured with an ion chamber array). The radiation fields shaped by ball bearing filled 3D printed cutout were centered within 0.4 mm of the planned outline, whereas the Cerrobend cutout fields had shift errors of 1–3 mm, and shape errors of 0.5–2 mm. The average shift of Cerrobend cutouts was 2.3 mm compared to the planned fields (n = 5). Beam penumbra of the 3D printed cutouts was found to be equivalent to the 15 mm thick Cerrobend cutout. The beam profiles agreed within 1.2% across the whole 30 cm profile widths.

**Conclusions:**

This study demonstrates that with a proper quality assurance procedure, 3D-printed cutouts can provide more accurate electron radiotherapy with reduced toxicity compared to traditional Cerrobend methods.

## Introduction

**Fig 1:**
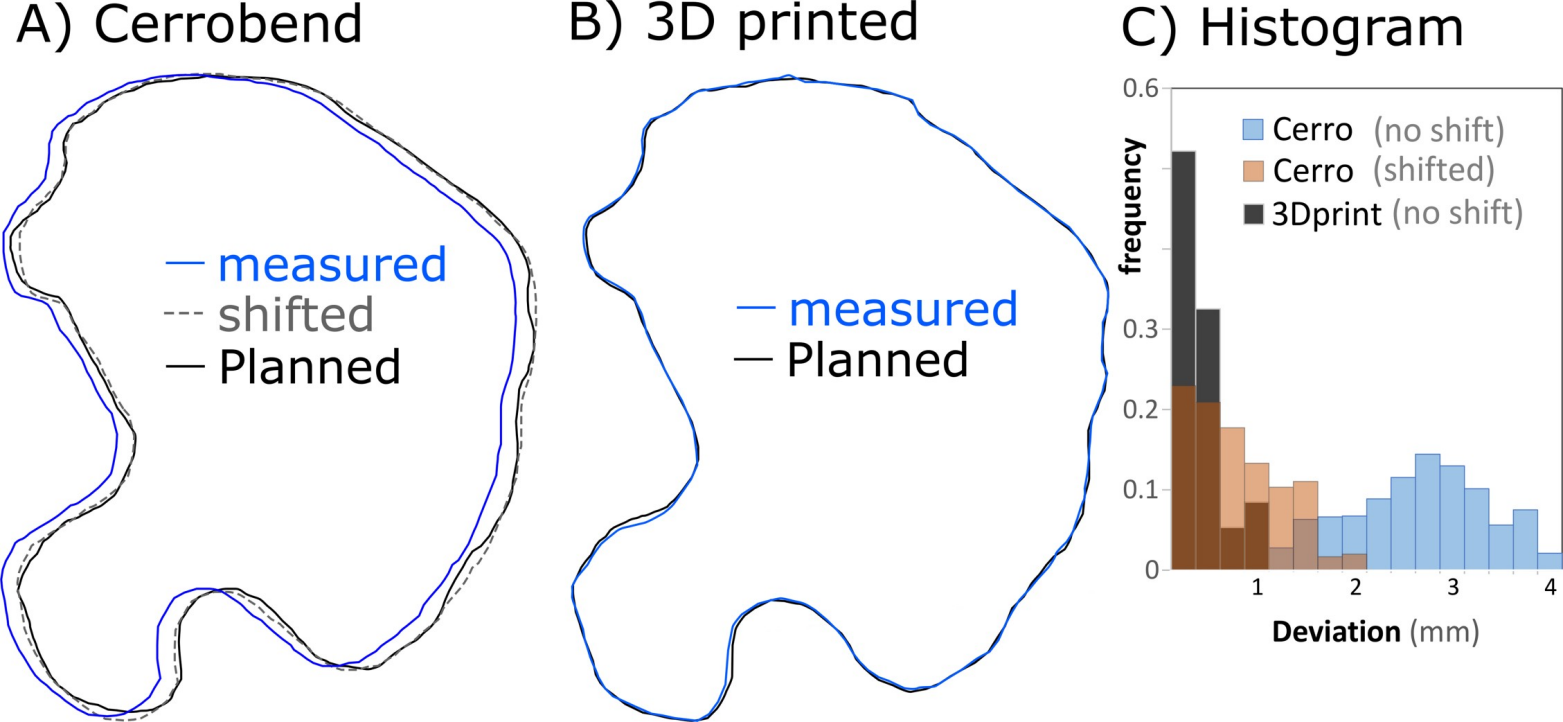
3D printed cutouts provide more accurate field shaping. (A) The planned (black line) and the measured Cerrobend outlines (blue line) have a mean deviation of 2.6±0.2 mm. Even after shifting, the Cerrobend outline (dashed line) shows a maximum deviation of 2 mm and a mean of 0.8 mm compared to the planned outline. (B) The 3D printed cutout (blue line) follows the planned outline (black line) more closely with maximum deviation of 1 mm and a mean of 0.4 mm. (C) Histogram of deviations between final and planned field shapes.

It has been long recognized that custom field shaping blocks used for electron beam therapy complicate and slow down the treatment procedure[1]. Although low cost, production of blocks cast from the low melting point alloys require manual labor and handling of toxic materials. In addition, transferring the outline from the treatment planning system (TPS) to the cutting tools introduces field shape and placement uncertainties of several millimeters. As an example of this error, Fig 1A shows the light field edge from a typical clinical Cerrobend insert compared to the planned outline. The 3D printed cutout methods in this study represent one way to reduce these uncertainties. While many current electron treatments do not require increased accuracy, developments in combination with modern dose calculations and modulating bolus [2–4] open up the potential for more advanced therapies, such as mixed beam therapy, which offer increased distal sparing compared to traditional intensity modulated, or volumetric arc radiation therapy photon plans [5, 6].

There are two major reasons for the inaccuracy of the Cerrobend cutout shown in Fig 1. First is the placement error: a foam casting block is manually placed to form the Cerrobend. Second is that the melt casting and cutting of the foam block themselves include some imprecision. In contrast, the 3D printed cutout is designed digitally, and its manufacture does not include placement error, manual cutting or melt casting. The placement inaccuracy, in particular, is significant as it limits accurate alignment of the electron field with the kV imaging x-rays and MV treatment x-rays. A major direction toward improving the accuracy of electron radiation therapy has been to develop electron multi-leaf collimators (MLC) [1, 7, 8]. While able to replace Cerrobend and provide intensity modulation, these add significant weight and complexity to the linear accelerator. Electron MLCs also require extra quality assurance (QA), cost, and maintenance. 3D printed cutouts, however, offer the possibility to improve electron radiation therapy with minimal additional cost, and without upgrading existing equipment or the treatment planning software.

While the recent improvements of fused deposition modelling (FDM) 3D printers have resulted in wide adoption of 3D printed bolus for radiation therapy [2–4, 9], less work had been published on 3D printing applications for shielding devices. This is largely because most printable plastic, or plastic-metal composites are limited in maximum density to 4 g/cm^3^. One solution is to use a three step process: (i) 3D print plastic parts, (ii) pour silicone or plaster to create molds, (iii) cast Cerrobend parts into the mold [10, 11]. This multistep process, however, is labor intensive and results in lower accuracy than directly using 3D printed parts. In this study, we took a simpler approach to achieve parts with densities above 10 g/cm^3^ with the use of a combination of 3D printed shells and tungsten filler material. A procedure of QA was developed to ensure the density of the shielding device.

## Methods and methods

### Cutout design and production

**Fig 2:**
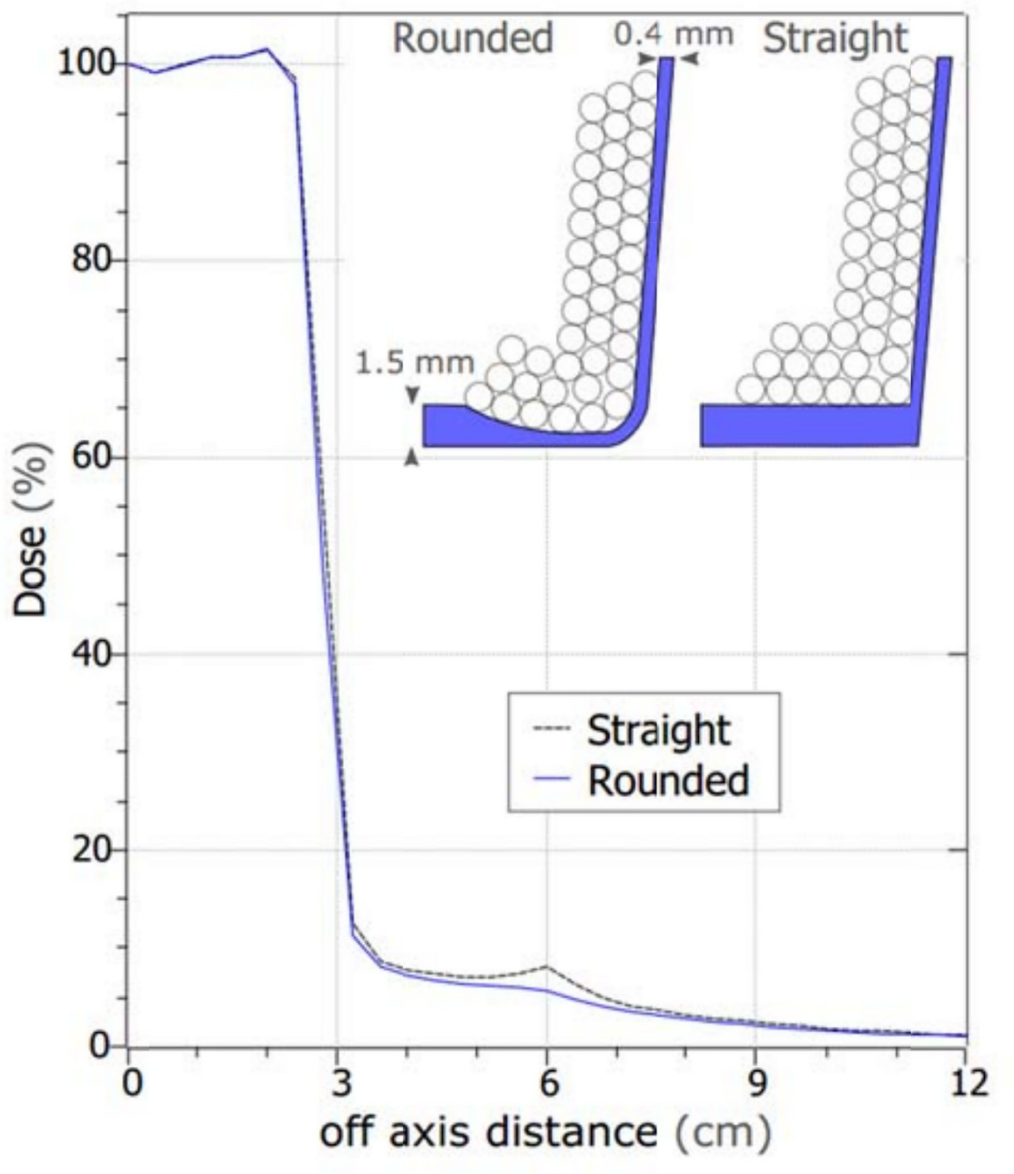
Comparison of 3D printed inserts with different cross section. Straight edge (right), with constant thickness bottom edge consistently produced worse dose tail (dashed line) than rounded edge (left) with thinned bottom edge close to the field (solid line). The slopes are to match the beam divergence. For clarity, only some spheres are drawn. Other off-axis directions have the same dose profiles. The 2–8% dose in the out of field regions is comparable to that for Cerrobend cutouts (see Fig 3, section 3 for further discussion).

**Fig 3:**
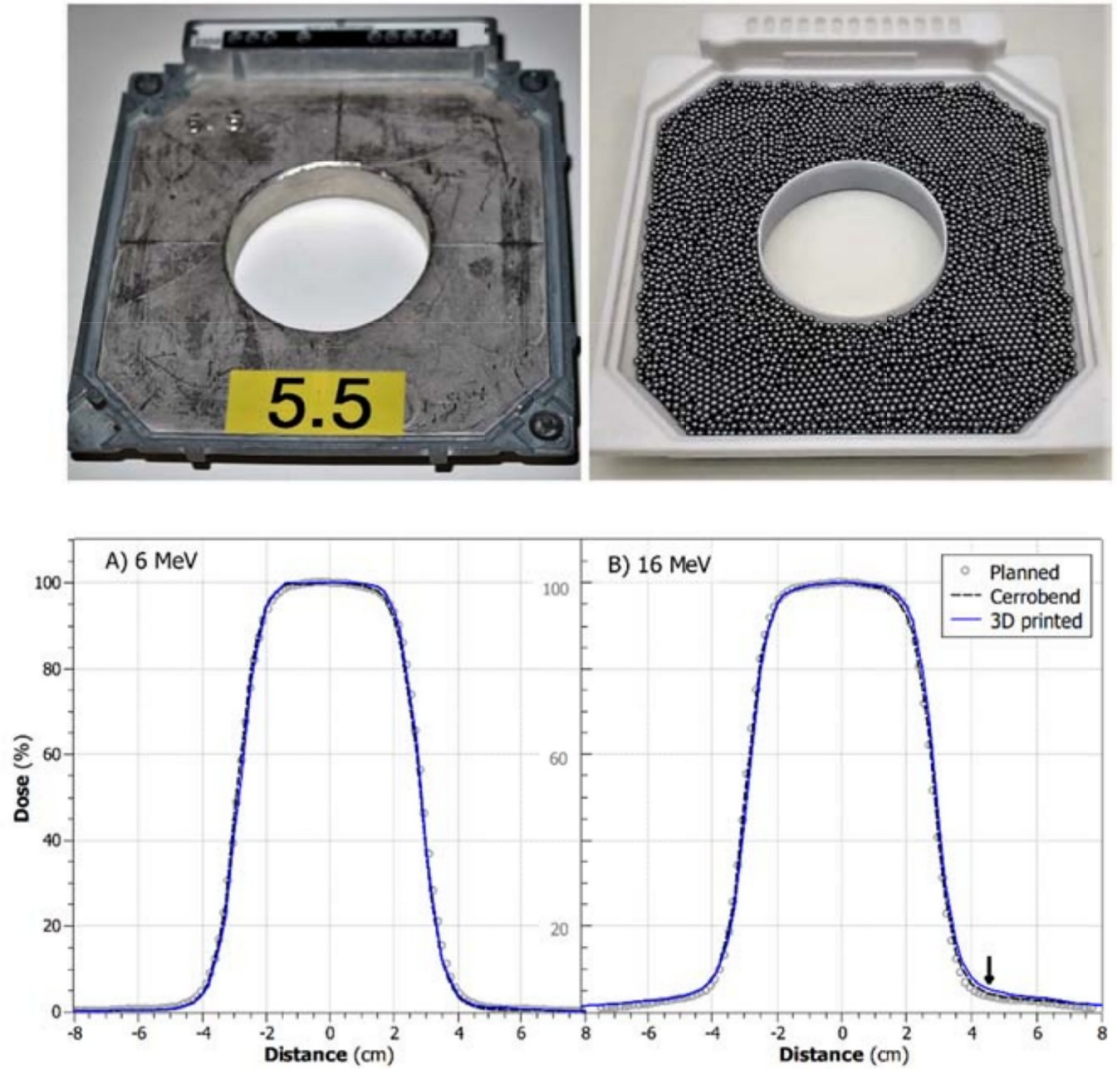
Upper panel: the 5.5 cm Cerrobend cutout (left) and 3D printed cutout (right). Lower panel: Crossline dose profiles across 5.5 cm circular inserts measured at a 0.9 and 2.7 cm water equivalent depths with (A) 6 MeV and (B) 16 MeV, respectively. The dose profiles of 3D printed insert (blue line) are centered better (0.1 mm and 0.3 mm for 6MeV and 16MeV, respectively) than that of Cerrobend insert (dashed lines, 0.7±0.1 mm for both energies). For 6 MeV both inserts show <1% dose at 5 cm off-axis. For 16MeV the planned dose (grey circles) is lower outside the radiation field than that of the measurements. The arrow on plot (B) highlights the dose at 5cm off-axis which is 4.1%, 3.5%, and 3.2% for the 3D printed insert, Cerrobend insert, and planned dose respectively. FWHM of the 3D printed, Cerrobend, and planned dose profiles are comparable with both energies. The circle was 5.5cm at 95 SSD.

3D printed plastic shells were designed to fit electron applicators (Varian, Palo Alto, CA), using freely available CAD software (TinkerCAD, Fusion360, inkscape), and printed with a commercial 3D printer (Ultimaker 2+). The printing material was polylactic acid (PLA) plastic. PLA was chosen for printing material due to its abundance, ease of use, and low cost for rapid prototyping. The plastic shells were filled with 2 mm diameter tungsten alloy ball bearings (THPP, San Diego CA) with a nominal density of 17.5 g/cm^3^. Tungsten alloys were chosen over other materials due to their high electron density, low toxicity, and reasonable cost (see Table 1 and discussion for further detail). To minimize the amount of scatter from the plastic, the bottom edge of the insert is designed to be thin in the areas close to the field (Fig 2). The dose profiles shown in Fig 2, including the Bremsstrahlung tails outside the field, are comparable to Cerrobend cutouts. See Fig 3, section 3 for direct comparison and further discussion.

**Table 1:** Physical properties of common materials. PMMA also known as acrylic or Lucite. Woods metal also known as Cerrobend. The “particle ρ” column represents the bulk packed density of that material in a powder or ball bearing form. Relative electron density is the number of electrons per unit volume relative to water.

material	Composition	<z>	Relative Bremsstrahlung	Relative electron density	Solid ρ g/cm^3^	particle ρ g/cm^3^
Tungsten alloy	W_24_Ni_4_Fe_2_	64	0.90	9.67	17.5	10.5
Lead	Pb	82	1.15	5.98	11	-
Woods metal (Cerrobend)	Bi_19_Pb_10_Sn_9_Cd_7_	71	1	5.08	9.7	-
Brass	CuZn	29.5	0.42	5.47	8.96	5.4
Steel	Fe_3_C	21	0.30	5.05	8.05	4.8
Al2O3 (Ceramic/Sapphire)	Al_2_O_3_	10	0.14	2.60	3.95	2.37
PLA (plastic)	C_3_H_4_O_2_	4.2	0.06	1.03–1.2	1.2–1.4	-
PMMA (lucite)	C_5_O_2_H_8_	3.6	0.05	1.07	1.18	-

**Fig 4:**
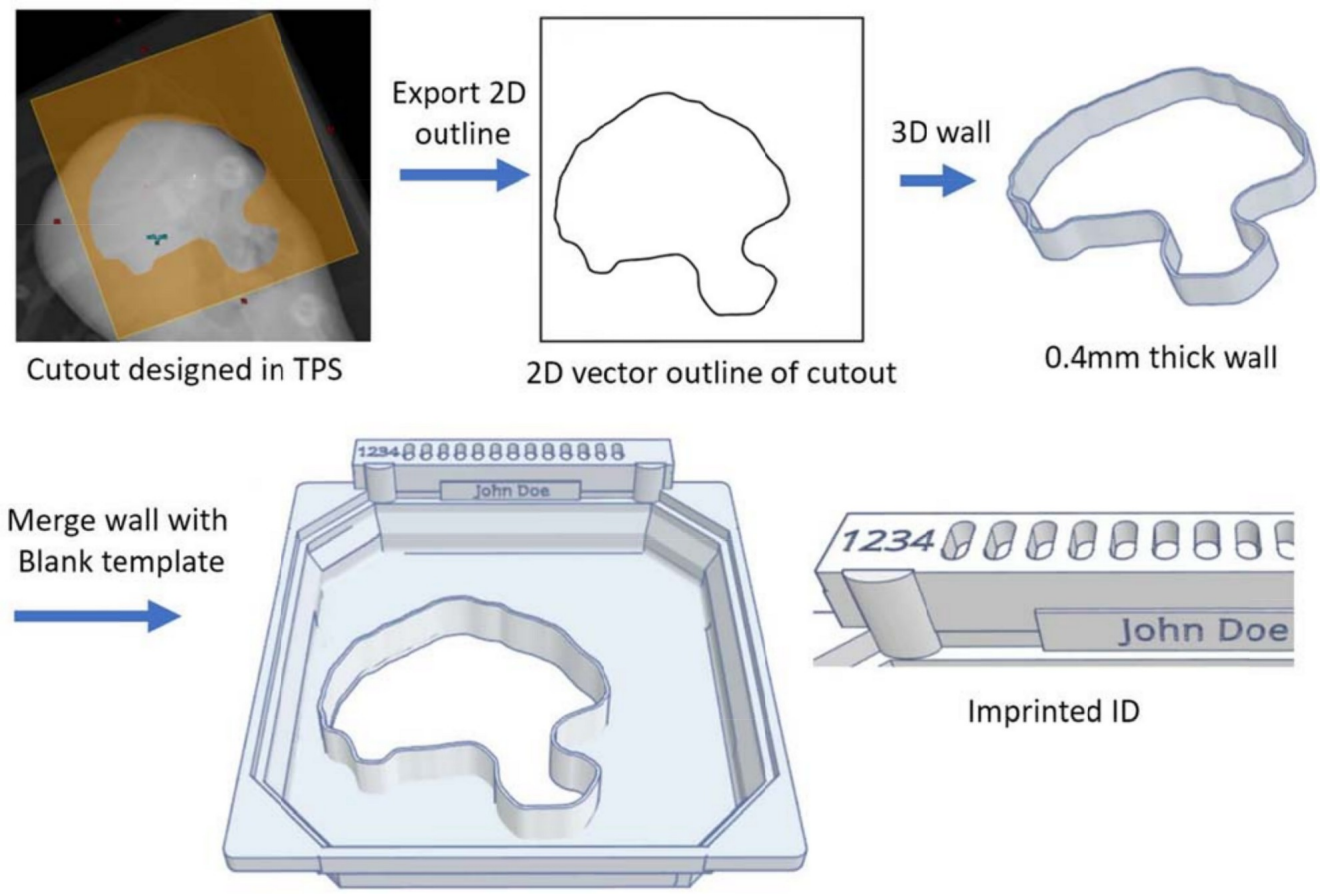
Workflow for the 3D printed cutout. First the field outline is exported from the treatment planning system and converted into a 2D vector image. The 2D outline is then imported in to CAD software and extruded into a 3D wall. Divergence and edge rounding fillets is also included in this step. The field edge wall is then combined with the template outline. Patient identifiers and custom codes can be imprinted directly into the final part.

Production workflow of the cutouts is streamlined by using a blank digital template for each applicator size. The template (Fig 4) consists of the encoder strip and tray insert shape that mates with the applicator cone. To create the single custom part, the template is digitally aligned and merged with the desired field outline shape. The field outline was generated by exporting from the Eclipse TPS (Varian, Palo Alto, CA, USA) into a pdf file. Instead of creating a physical printout as for Cerrobend, the outline was digitally traced and saved as a 2D vector file. This 2D outline was then taken into 3D design software (Autodesk fusion360, San Francisco, CA, USA) expanded and extruded to create a thin wall of 0.4 mm in thickness and 15 mm in height (3 mm of which are taken by the base and lid). This field shape part is then digitally aligned and combined with the blank tray template into a single part. Patient ID, name, and custom codes were also 3D printed into the part to avoid using the wrong block for the patient to improve safety. The workflow for the 3D printed cutout is shown in Fig 4.

To compare the field shape and placement of Cerrobend cutouts to the planned fields, five on-treat clinical Cerrobend cutouts were placed in the electron applicator mounted on to a linear accelerator (Varian, Palo Alto, CA, USA). The light field edges shown in Fig 1 were compared to the planned outlines on printed paper using the following method: The printout was placed on the couch at 110 cm Source to Surface Distance (SSD) and aligned to the square light field with no insert. The insert was then placed in the applicator and the light field edge from the cutout was traced onto the printout, which also contained the planned outline. The printout and tracing were then digitized, and the shift required to minimize the difference between the planned and traced outlines was measured.

### Dosimetric evaluation

Two field shapes were evaluated. Namely a 5.5 cm diameter circle, and an anonymized clinical treatment field. In both cases, 3D printed inserts were created and filled with 2 mm diameter tungsten ball bearings to a depth of 12 mm. These were compared to Cerrobend cutout inserts of the same shapes, which were 15 mm thick.

An ion chamber array (IC Profiler, Sun Nuclear, Melbourne, FL, USA) was used to measure dose profiles of the cutouts with 6 MeV and 16 MeV. To increase the resolution, a second exposure was taken with a 2 mm shift in each profile direction. This provided data points every 2 or 3 mm from the 5 mm spaced array. The detector was centered using the light field cross hair of the linear accelerator. The water equivalent measurement depths were 0.9 cm and 2.7 cm for 6 MeV and 16 MeV, respectively with 100 cm SSD. 300 monitor units (MU) were delivered for each profile measurement, using a Varian Clinac 21EX linear accelerator (Varian, Palo Alto, CA). The measured dose profiles were then compared to the TPS using the eMC algorithm (Varian eclipse v13.7, Palo Alto, CA), by importing the dose plane into the profiler software. Centering and full width half maximum (FWHM) metrics were taken directly from the profiler software.

For the clinically relevant field shape, 16 MeV, 300 MU dose profiles were measured with an IC profiler ion chamber array at a water equivalent depth of 2.7 cm and an SSD of 110 cm on a Varian 21EX linear accelerator. The same 2 mm shift procedure described above was used to increase the spatial resolution.

### Quality assurance

A QA procedure was developed to ensure the cutout is correctly filled and printed. The printed insert is first visually inspected to make sure there is no major defect. The proper tungsten ball bearings filling is measured the by weight of the cutout using Eq (1)

$$W_{tot}\geq V_{BB}∙{pf∙\rho }_{BB}+W_{ins}.$$ (Eq 1)

where *W*_*tot*_ is the expected weight, *V*_*BB*_ is the volume of the insert available for the ball bearing filler, obtained from the 3D files, *ρ*_*BB*_ is the density of the tungsten ball bearings (17.5 g/cm^3^), *pf* is the packing fraction and *W*_*ins*_ the weight of the insert. Here we use the minimum acceptable *pf = 0*.*6*.

The weight, *W*_*tot*_, was measured using a digital scale calibrated using standard weights to within 0.1%. To pass the QA, the measured weight should satisfy Eq 1. Field shape verification was performed by overlaying the cutout with a transparent printout from the TPS on transparent paper to compare the shape of the cutout. The 3D printing process, standard printing profiles and procedures should be used to ensure consistency. Attention should be paid to the 1^st^ layer of each print as if this does not fully stick to the print bed it can cause distortion.

## Results

Dose profiles of 6 MeV and 16 MeV electron beams delivered through the 5.5 cm circle of Cerrobend and 3D printed cutout are shown in Fig 3 (10 x 10 cm^2^ insert). The planned, Cerrobend, and 3D printed dose profiles (80% to 20% penumbra widths) agree within 0.4 mm. The comparisons between the FWHM, centering, and off-axis dose of the planned, Cerrobend and 3D printed cutouts are listed in Table 2. The 5.5 cm cutout circle was defined at 95 cm SSD such that it produces field sizes of 5.8 cm in diameter at 100 cm SSD. The FWHM for 3D printed circle, and the planned dose profile agreed within 1 mm. The centering of the Cerrobend circle was found to be up to 0.7 mm off center, compared to 0.1 mm and 0.3 mm for the 3D printed circle at 6 MeV and 16 MeV, respectively. The 12 mm deep volume is calculated to be 159.7 cm^3^. The tungsten ball bearings were weighed to be 1652 g which is giving a density of 10.34 g/cm^3^. This is in close accordance with the expected density from random sphere packing theory (17.5 g/cm^3^*0.6 = 10.5 g/cm^3^). The dose under the block was up to 0.9% higher for the 3D printed cutout at 16 MeV than that for the planned dose or Cerrobend cutout (Fig 3 and Table 2).

**Table 2:** Measured centering and width of profiles from a Sun nuclear profiler 2 detector aligned to light field crosshairs on a Varian True beam linear accelerator. For comparison, the planned dose plane was imported and compared in the same profiler software.

	6 MeV			16 MeV			Physical Size Measurements	
Insert type	FWHM (cm)	Center (cm)	Dose 5cm off axis	FWHM (cm)	Center (cm)	Dose 5cm off axis	Insert Average diameter (cm)	diameter variation (cm)
3D printed	5.70	0.01	0.95%	5.77	-0.03	4.1%	5.46	0.03
Cerrobend	5.71	-0.07	0.8%	5.78	-0.06	3.5%	5.44	0.08
planned	5.77	N/A	0.50%	5.77	N/A	3.2%	N/A	N/A

**Fig 5:**
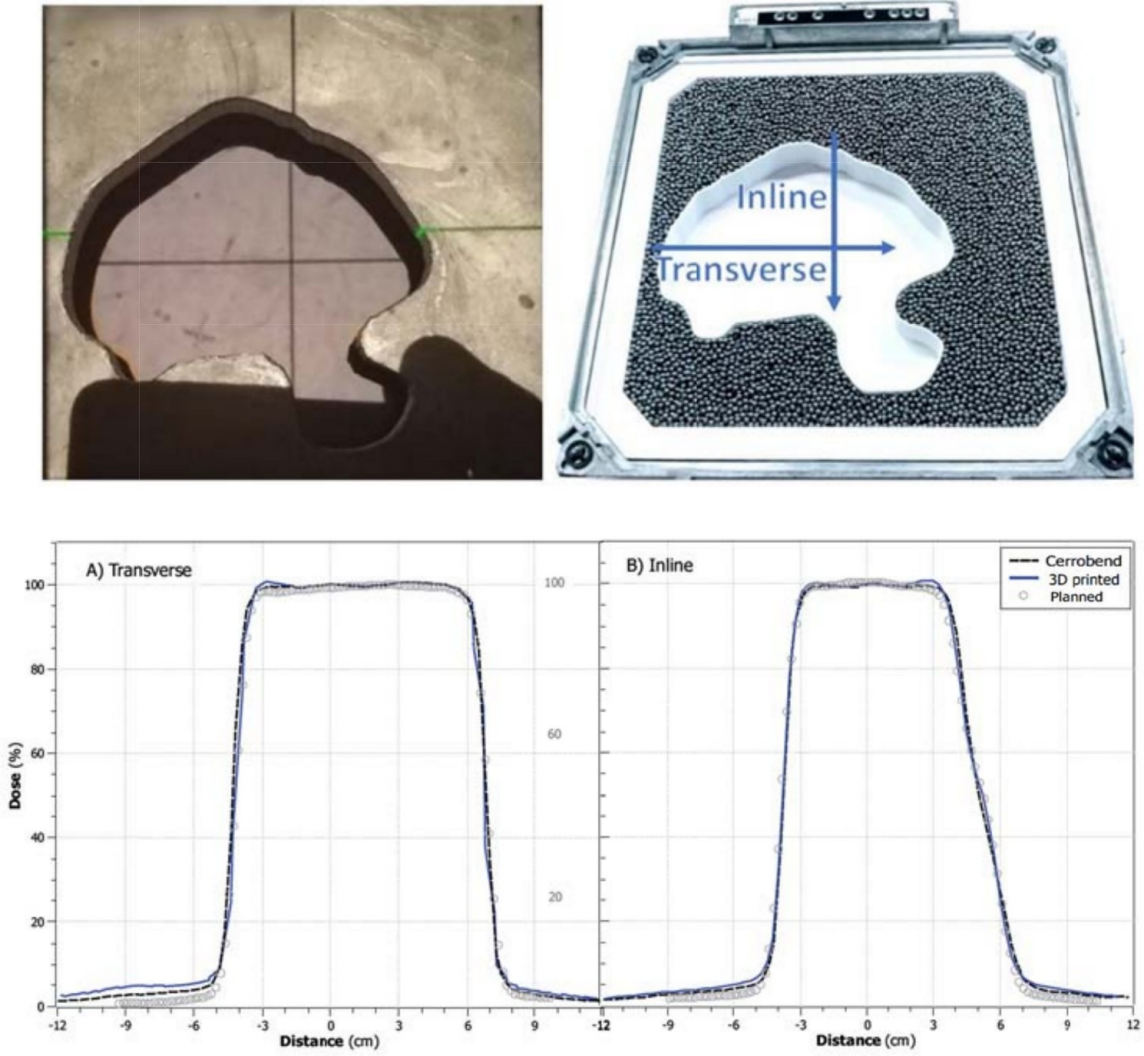
Upper panel: Cerrobend (left) and 3D printed (right) cutouts. Lower panel: Dose Profiles of 16MeV beam through the Cerrobend (dashed line) and 3D printed cutouts (solid line) measured with an IC profiler array at a 2.7 cm water equivalent depth. The planned dose is the dose in a water phantom calculated from the eclipse treatment planning system using the eMC dose calculation algorithm (circles).

A Clinically representative field shape is shown in Fig 5. This field was reproduced with both Cerrobend and 3D printed cutouts. The measured profiles with a 16 MeV beam were aligned to correct for the 2.7 mm shift present in the Cerrobend block compared to the treatment plan (Fig 1). The measured Cerrobend and 3D printed profiles agreed with each other within 1.5% or 1 mm for all points above 10% of the maximum dose.

## Discussion

A simple and non-toxic solution for electron field shaping using 3D printing and tungsten ball bearings is presented. By replacing Bismuth-lead alloy block casting, this method reduces manual labor and removes toxic materials from the clinic. Safety is also improved through printing of patient’s information and custom codes directly into the 3D printed part. This method allows accurate field shaping using standard applicators. The all-digital workflow ensures accuracy and reproducibility of the inserts.

A major consideration with using filling powder or ball bearing materials is the packing fraction, and its effects on density. Whether filling a volume with powder or ball bearings, the hard sphere random packing fraction concepts are scale invariant and remain essentially the same. For monodisperse (same radius) spheres the highest possible random packing fraction (the fraction of filled volume to total volume) is around 0.64 [12, 13], with a packing fraction of 0.6 being an easily achievable value. Here, we investigate tungsten alloy ball bearings and powders for their potential to achieve high electron density at low cost. Using tungsten alloy ball bearings (17.5g/cm^3^) with a packing fraction of 0.6 yields a bulk density of 10.5 g/cm^3^. This way a low cost shaped block with a density comparable to lead, without toxic materials, and approximately 10% less Bremsstrahlung than Cerrobend can be achieved (see Table 1, relative Bremsstrahlung is estimated from the ratio of the mean Z^2^ compared to Cerrobend).

Of the non-toxic materials with higher electron densities than tungsten (z = 74) there is only rhenium, osmium, iridium, platinum, and gold (z = 75–79). All of which are prohibitively expensive. Elements with z = 80 (mercury) or greater were excluded due to either chemical toxicity or radioactivity.

Materials with lower electron density may also be considered. Machined Brass, for example, is sometimes used due to it having comparable electron density to lead, but with lower z, and hence lower Bremsstrahlung production. There are two main drawbacks to using lower density materials for electron field shaping: (i) the increased cutout thickness results in broader penumbra, and (ii) the lower x-ray absorption of lower density materials means they are less able to shield Bremsstrahlung x-rays produced up stream of the cutout, or those generated in the cutout itself. When considering Bremsstrahlung it is important to note that, 70–90% of the Bremsstrahlung of a typical electron beam therapy is generated in the linac head (e.g. scattering foils), not the final Cerrobend aperture [14]. This means that the ability of the cutout to absorb head generated bremsstrahlung should be considered as well as the Bremsstrahlung that the cutout itself generates. The lower relative Bremsstrahlung (Table 1) of tungsten alloy compared to Cerrobend, due to its lower z, demonstrates that it should produce less Bremsstrahlung than standard Cerrobend cutouts.

Measurements show comparable dose profiles for tungsten ball bearing filled insert verses the standard Cerrobend insert for energies up to 16 MeV. This density was achieved with no attempt to maximize the number of ball bearings in the volume, they were poured in without any pressing, or rearrangement. The 16MeV dose measurements given in Table 2, and profiles plotted in Figs 3 and 5 show slightly increased out of field dose. The dose at 5 cm off axis is 4.1%, 3.5% and 3.2% of the maximum dose for the 3D printed insert, Cerrobend insert, and planned dose respectively. The most likely cause of the small increase is electron scatter from the plastic walls of the 3D printed cutouts. This could be mitigated by 3D printing a dense metal shell or by making thinner plastic walls. One trick to obtain higher densities is to fill the space between the ball bearings with powder or fluid. Then bulk average densities of 14.7 g/cm^3^ (10.5*0.4+17.5*0.6) can be achieved. This double-filled option was not investigated here due to the added complexity and clean up required for the clinical workflow.

The 16 MeV maximum energy investigated in this work was chosen as this is frequently used clinically. While many linear accelerators are capable of producing higher electron beam energies, photon or proton treatment modes are normally preferred to reach deep seated tumors that require 20 MeV and higher electron beams. Since the collisional stopping power of tungsten only varies between 1.02 and 1.36 MeV cm^2^/g in the range 1-50MeV, the cutout thickness required to shield higher energy electron beams increases approximately linearly with energy.

The 3D printed shell can be optimized to print in 2–5 hours, on standard commercial 3D printers. While slightly slower than Cerrobend, which can be made within in one hour, the 3D printing process requires only approximately 5–15 min of labor to fill, clean up and verify the printed cutout. One concern of using PLA-based 3D printed shells is its strength, fracture and toughness [15]. Designs produced here were found to be robust and can be easily modified to add strength as needed. If desired, stronger, and more heat resistant plastics such as carbon fiber reinforced nylon, or polycarbonate or polyethylene terephthalate (PET) based plastics can just as easily be 3D printed. With more time and investment cost, metal 3D printed shells are also possible. A benefit of 3D printed parts is that if they break, they can be accurately reproduced within a few hours with minimal additional labor. Other choices could also be made for the filler material. However, little to no benefit is foreseen going to lower z or lower density materials; this would necessitate a thicker cutout and would less effectively block Bremsstrahlung from the linear accelerator’s head [16, 17]. One unforeseen benefit was that the 3D printed insert is more reliably read by the Varian code reader than the standard inserts; the 3D printed tray is a single part and is made to tighter tolerances. In our clinical practice the inconsistency of the Varian code readers and inserts causes treatment delays and inconvenience to patients. The imprinted name on the block also provides an intuitive way for the therapists to check the cutout before the treatment (Fig 4).

## Conclusion

In this work 3D printed designs for electron cutout have been demonstrated to accurately reproduce the dose profiles compared to that of Cerrobend cutouts. This method removes toxic material from the clinic, reduces manual labor, and provides improved reproduction of the field placement and field shape compared to Cerrobend. One caveat is that for higher energies the thickness of the cutout may need to be increased. Given the current rapid rate of development of 3D printing, it is expected that these technologies will be dramatically improved in the coming years, giving yet more convenience, speed, and precision. This increased precision, in concert with other recent developments, such as modulating electron bolus, opens up new opportunities for advancing electron radiotherapy.
